# Supplementary material for: Drug repurposing for aging research using model organisms
Source: Aging Cell. 2017 Jun 16;16(5):1006–15. doi: 10.1111/acel.12626 (PMC5595691; doi:10.1111/acel.12626)
Supplement: Supplementary file 7 — Data S1 Zip‐Archive of all report cards. [file ACEL-16-1006-s007.zip › RC_LHL.pdf]

LHL

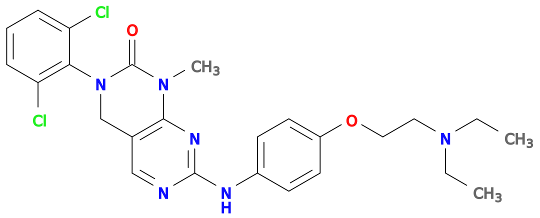

Database identifiers

ChEMBLCompound	CHEMBL1234002

Ranking

	Rank	Score
Drosophila	270/697	0.617
C. elegans	NA	NA

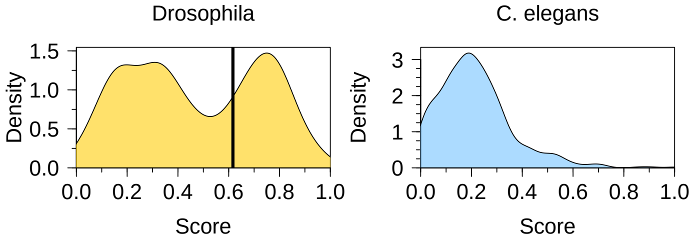

	Ageing implication	Domain conservation	Binding site conservation	Binding affinity	Bioavailability	Lipinski	Promiscuity	Purchasability	Drug approval	**Total**
Drosophila	1.0	0.937	0.882	0.965	(0.9)	-0.1	-0.0	0.0	0.0	0.617
C. elegans	NA	NA	NA	NA	NA	NA	NA	NA	NA	NA

Names

No synonyms found

Roles

ChEBI entry None has no roles

Status

Approved drug (according to ChEMBL)	No
Number of Rule of 5 violations	2
Binding affinity to original target in log units (RF-Score prediction)	8.31
Burns *C. elegans* bioavailability prediction	3.27

Compound Target Characteristics

Tyrosine-protein kinase BTK

Best gene implication in ageing for this target family came from gene P08630 annotated in UniProt release 2014_02. Annotation GO 8340 (determination of adult lifespan) was Inferred from Mutant

Phenotype

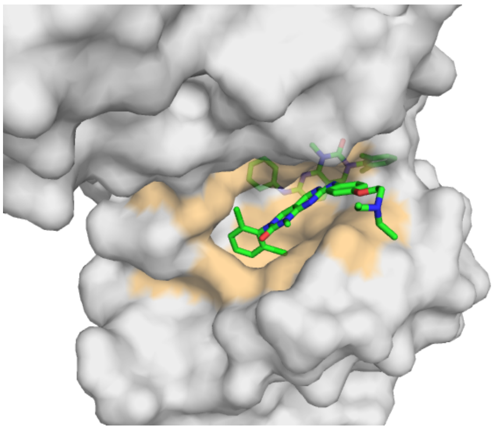

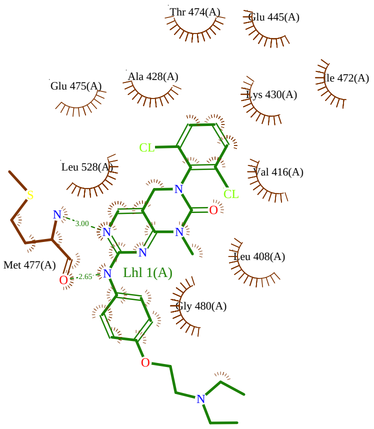

protein	amino acids contacts (binding site)
PDB:3pj1:chainA:Q06187	L V Y A I K E S Q Y I T E M G L S D
tr:Q3MS96:Q3MS96_HUMAN	L V Y A I K E S Q Y I T E M G L S D
sp:Q06187:BTK_HUMAN	L V Y A I K E S Q Y I T E M G L S D
tr:Q501W1:Q501W1_RAT	I V I A I K E S Q Y I T E M G L S D
tr:B2RYC7:B2RYC7_RAT	L V Y A I K E T Q Y I T E M G L S D
sp:P35991:BTK_MOUSE	L V Y A I K E S Q Y I T E M G L S D
sp:P08630:BTKL_DROME	L V I A V K E Q Q Y I T E M G L A D

	whole protein		domain-based		contact-based	
protein	ident	simil	ident	simil	ident	simil
PDB:3pj1:chainA:Q06187	1.0	1.0	1.0	1.0	1.0	1.0
tr:Q3MS96:Q3MS96_HUMAN	0.48	0.48	0.96	0.96	1.0	1.0
sp:Q06187:BTK_HUMAN	1.0	1.0	1.0	1.0	1.0	1.0
tr:Q501W1:Q501W1_RAT	0.4	0.65	0.62	0.88	0.89	0.98
tr:B2RYC7:B2RYC7_RAT	0.52	0.8	0.64	0.89	0.94	0.97
sp:P35991:BTK_MOUSE	0.98	1.0	0.99	1.0	1.0	1.0
sp:P08630:BTKL_DROME	0.36	0.63	0.58	0.87	0.78	0.88

Btk29A (FBgn0003502) associated phenotypes

cell shape defective, lethal - all die before end of first instar larval stage, mating defective, partially, short lived, size defective, some die during first instar larval stage

(Information from FlyBase)

Btk29A (UniProt:P08630) annotation

Function: Required for proper ring canal development. Also required for the development of male genitalia and for adult survival. (PubMed:10330180, PubMed:9655810).
Cofactor: Zn(2+)Note=Binds 1 zinc ion per subunit. ;
Subcellular location: Note=Ring canals.
Tissue specificity: Ring canals in the egg chambers and imaginal disks of third-instar larvae. (PubMed:10330180, PubMed:3110602, PubMed:9655810).
Developmental stage: Expressed both maternally and zygotically. Predominantly in early to middle embryogenesis, in larvae and adult females. (PubMed:10330180, PubMed:3110602).
Disruption phenotype: Flies exhibit shortened copulatory duration (due to incomplete fusion of the left and right halves of the apodeme that holds the penis during copulation) and reduced adult-stage life span. (PubMed:10330180).

(Information from UniProt)
